# Supplementary figures and images for: MLKL deficiency protects against low-grade, sterile inflammation in aged mice
Source: Cell Death Differ. 2023 Feb 8;30(4):1059–71. doi: 10.1038/s41418-023-01121-4 (PMC10070424; doi:10.1038/s41418-023-01121-4)

Supplementary Figure 1C

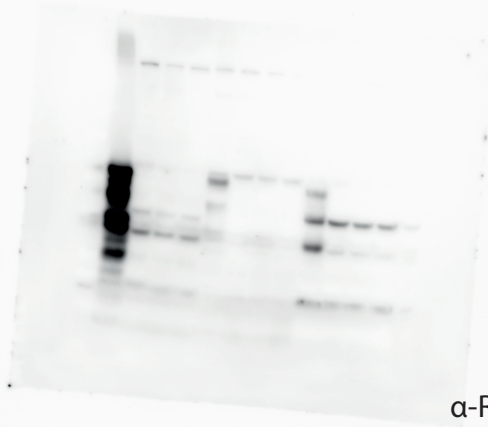

$\alpha$ -RIPK3

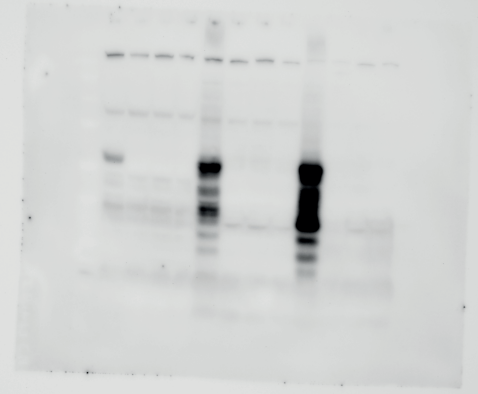

$\alpha$ -RIPK3

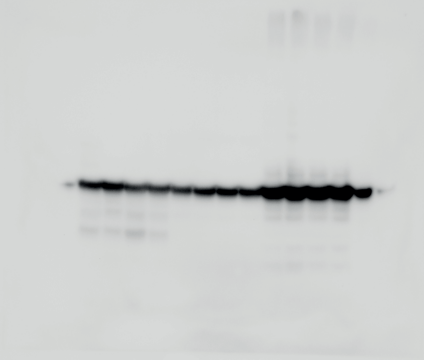

$\alpha$ -GAPDH

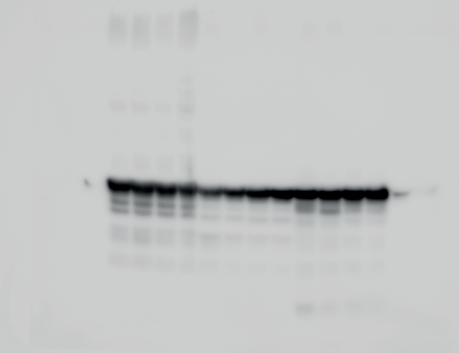

$\alpha$ -GAPDH

Supplementary figure 1E

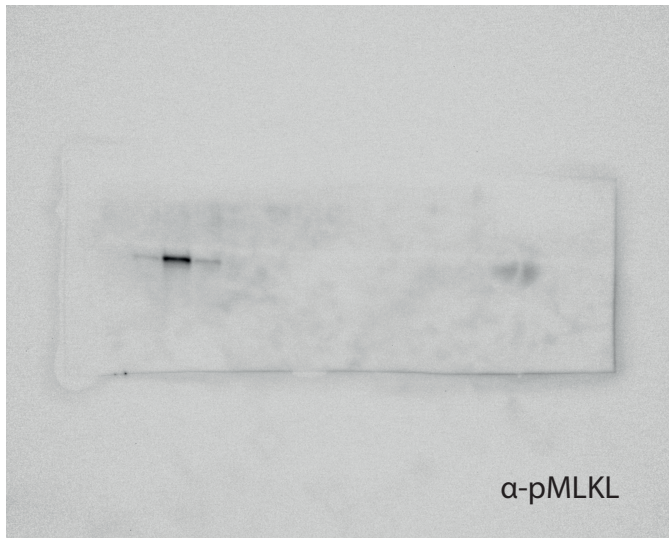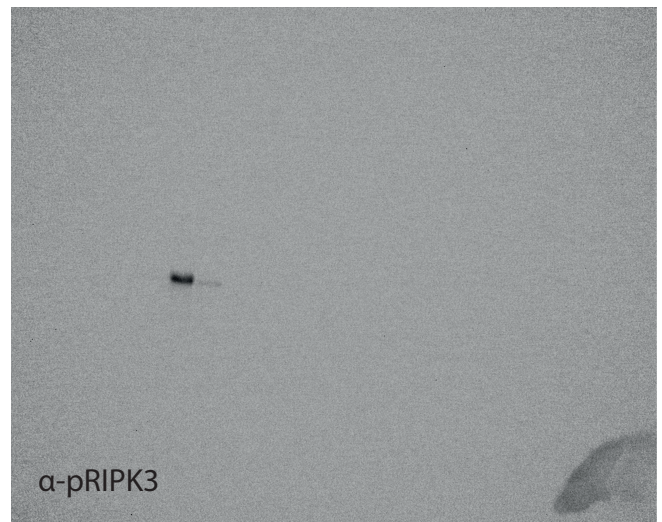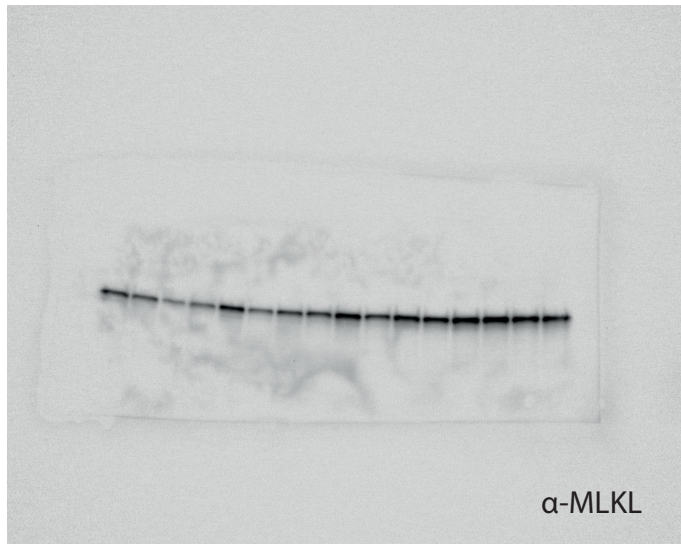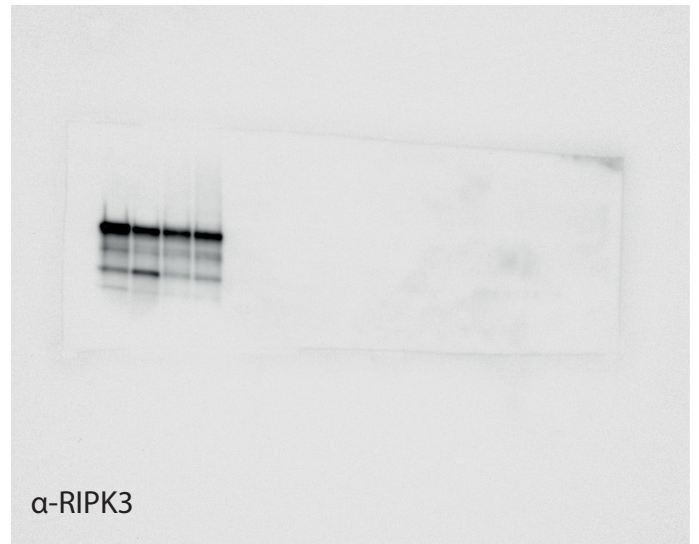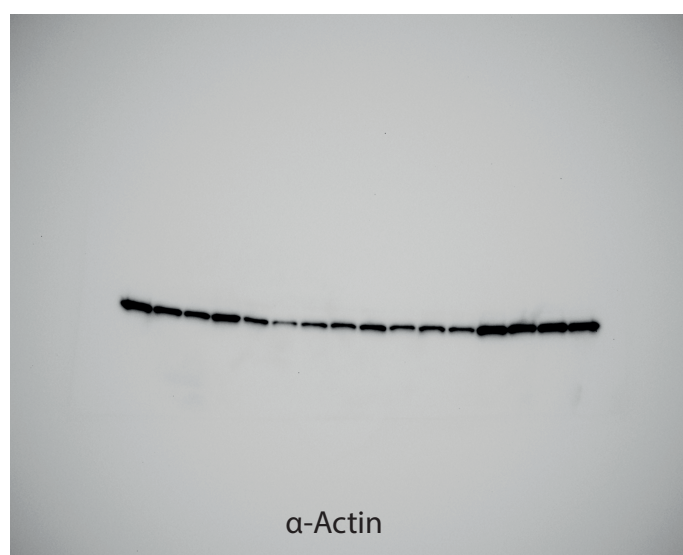

Supplement: Supplementary file 4 — Original Data File [file 41418_2023_1121_MOESM4_ESM.pdf]
